# Identification of Amino Acid Conservation in The Curli Accessory Protein CsgF

**DOI:** 10.17912/micropub.biology.001702

**Published:** 2025-07-16

**Authors:** Karen Guerrero, Emma Smith, Shruti Sunder Rajkumar, Zachary Cairo, Jonathan Adame, Renad Rawas, Ranim Rawas, Sajith Jayasinghe

**Affiliations:** 1 Chemistry and Biochemistry, California State University, San Marcos, San Marcos, California, United States

## Abstract

The Curli-Specific gene product F (CsgF) plays an important role in the assembly of gram-negative bacterial cell surface filaments known as Curli. In order to evaluate amino acid conservation in the context of the solution and CsgG bound structures of CsgF we carried out a multiple sequence alignment of CsgF sequences from 35 gram-negative bacteria and correlated amino acid conservation to structural and functional importance. We identified conserved Pro and Gly residues within the N-terminal region of CsgF that may be required to adopt the loop confirmation observed in this region. Several conserved hydrophobic residues are found on the 3rd and 4th β-strands of the C-terminal β-sheet and extending to the C-terminal end, that may play a role in the reported observation that the C-terminus is needed for Curli formation. The importance of several conserved residues that were identified in this study has not yet been reported and investigating their impact on the structure and function of CsgF may add to our understanding of Curli assembly.

**Figure 1. Amino acid conservation in CsgF:**
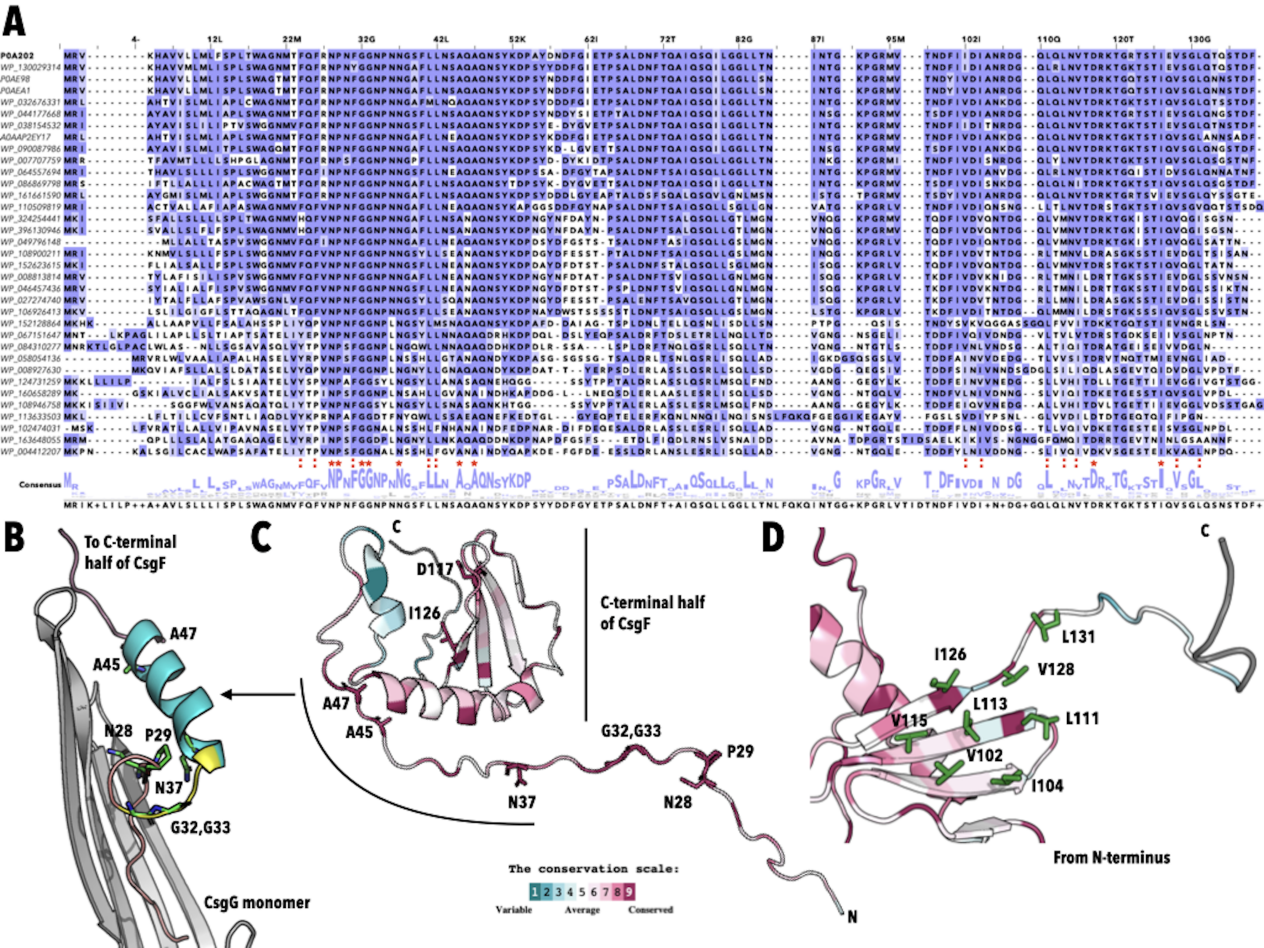
**A** Multiple Sequence Analysis (MSA) of CsgF sequences from 35 bacterial species (see extended data for details). Based on the MSA we identified nine residues, N28, P29, G32, G33, N37, A45, A47, D117, and I126, that are conserved in all 35 sequences (red star "*"). Red ":" indicate conserved residues that correlate with published descriptions of amino acids or regions of CsgF that are implicated as being important for its structure or function. **B** Structure of the N-terminal residues of CsgF in association with the Curli secretion channel CsgG (PDBID 6L7C and 6LQH). Only one monomer of CsgF and CsgG are shown. The N-terminal residues (20-31) of CsgF interact with the inner surface of the CsgG channel in an extended conformation ending in a loop (salmon). The loop extends between residues 29-36 of CsgF (yellow) and is followed by an 𝛼-helix that spans residues 37-46 (teal). Seven of the nine amino acids found in all 35 sequences, N28, P29, G32, G3, N37, A45, and A47, are found in this region (shown in stick representation). **C** Solution structure of CsgF (PDBID 5MIU) colored by evolutionary conservation as calculated by the ConSurf server where purple indicated high conservation and teal indicated low (see scale). All nine amino acids conserved in all 35 sequences are highlighted using stick representation. The region between positions 37-47 are in an extended conformation in the solution structure but adopt a helical structure in the CsgG associated form (Yan et al., 2020). **D** C-terminal 𝛽-sheet of the solution structure of CsgF colored by evolutionary conservation. The conserved positions containing amino acids with hydrophobic side chains are highlighted (green) and labeled.

## Description

The outer surface of gram-negative bacteria contain filaments, known as Curli, that play an important role in cell-cell interactions and host-cell colonization (Collinson et al., 1997, Sukupolvi et al., 1997, Austin et al., 1998, Römling et al., 1998). Curli are composed of two Curli specific gene (Csg) proteins, CsgA and CsgB, with CsgA being the major component of the Curli filaments (Chapman et al., 2002, Hammar et al., 1996, Bian & Normark, 1997). The assembly of CsgA/CsgB into Curli involves four other proteins: CsgC, CsgE, CsgF, and CsgG. CsgC and CsgE are periplasmic proteins that are thought to prevent the intracellular aggregation of CsgA/CsgB and target these proteins to the outer membrane channel CsgG (Gibson et al., 2007, Taylor et al., 2011, Evans et al., 2015). CsgA and CsgB traverse through CsgG and are secreted to the bacterial outer surface where they associate to form Curli (Cao et al., 2014, Goyal et al., 2014). In contrast, CsgF is located on the extracellular surface of bacteria and is required for Curli assembly (Robinson et al., 2006, Nenninger et al., 2009, Chapman et al., 2002). Structural studies have shown that the N-terminus of CsgF interacts with the CsgG pore, and in-vivo complementation assays have shown that the C-terminal half of the protein is required for proper Curli assembly (Zhang et al., 2020, Yan et al., 2020, Swasthi et al., 2023). A majority, if not all, of the structure/function studies reported so far have concentrated on *E. Coli* CsgF. We carried out a multiple sequence alignment (MSA) of CsgF sequences from 35 gram-negative bacteria (see extended data) to determine amino acid conservations and evaluated these in the context of the three-dimensional structures of CsgF to correlate amino acid conservation to structural and functional importance.

The MSA shows significant conservation throughout the sequence ( Figure 1A ). There are nine amino acids that are conserved in all 35 sequences (numbered according to the position in the *Salmonella* sequence): N28, P29, G32, G33, N37, A45, A47, D117, and I126 ( Figure 1A, red star "*"). We evaluated if these residues contributed to the interaction of CsgF with the CsgG channel using the cryo-EM structures of the CsgF-CsgG complexes available in the protein data bank (PDB ID 6L7C and 6LQH) (Zhang et al., 2020) (Yan et al., 2020). The N-terminal residues (20-31) of CsgF are observed to interact with the inner surface of the CsgG channel in an extended conformation ending in a loop ( Figure 1B, salmon). The loop which extends between residues 29-36 of CsgF ( Figure 1B, yellow) is followed by an 𝛼-helix that spans residues 37-46 ( Figure 1B, teal). Seven of the nine amino acids found in all 35 sequences, N28, P29, G32, G3, N37, A45, and A47, are found in the region that contains these three structural elements ( Figure 1B ). N28 (100% conservation) and N30, which is conserved in 74% of the sequences included in this study, forms H-bonds with E216 of CsgG suggesting that they may play a role in anchoring CsgF to CsgG. Yan et. al report a reduced interaction of CsgF with CsgG for the N28A variant but not for the N30A variant (Yan et al., 2020). The loop region contains P29, G32, and G33 which are found in all 35 sequences. These Pro and Gly residues, both of which are found in structural motifs requiring tight turns or flexibility, may play an important role in this region’s ability to adopt the loop observed in the CsgG bound form of CsgF. N37 which is conserved in all 35 sequences forms a H-bond with the backbone carbonyl of R27 and may also interact with the backbone carbonyl of P29 from an adjacent CsgF (which is 3.5 A away), and therefore may also play a role the formation of the loop conformation. As far as we know the impact of variants of these four residues have not been reported but would help in determining the influence of the loop region on the function of CsgG bound CsgF. A45 and A47, which are also found in all 35 sequences, are found on the C-terminal end of the 𝛼-helix that associates with the upper part of the CsgG pore. Again, no variants of these two alanine residues have been investigated and therefore, it is not known if these residues play a role in the association of CsgF with CsgG or its function.

Residues that have been identified as helping anchor CsgF to CsgG via van der Waals or hydrophobic interactions F24 (60% F, 34% Y, F26 (66% F, 34% Y, F31 (97% F, 3% Y), L41 (97% L) and L42 (91% L 6% F), are also conserved ( Figure 1A, red " **:"** ) (Zhang et al., 2020, Yan et al., 2020). Yan et. al report that the L41D and L42D variants of CsgF reduced its interaction with CsgG and that the F24D, F26D, and F31D, completely abolished the ability of CsgF to interact with CsgG (Yan et al., 2020) . These observations correlate with the conservation observed for these five amino acids.

The cryo-EM structure of the CsgF-CsgG complex does not contain the C-terminal residues of CsgF. In order to visualize the amino acid conservation in the context of structure we used the ConSurf server to map evolutionary conservation onto the three-dimensional solution structure of CsgF (PDBID 5M1U, Figure 1C ) (Yariv et al., 2023)(Schubeis et al., 2018) (Landau et al., 2005). We observe significant conservation throughout the solution structure of CsgF ( Figure 1C, purple) similar to what was observed via the MSA. In addition to conservation in the N-terminal half of the protein there is considerable conservation of residues in the 𝛼-helix and 𝛽-sheet found in the C-terminal half of the solution structure of CsgF. It has been observed that the 𝛽-sheet contains a hydrophobic surface formed from positions 102, 104, 113, and 115 on the 3rd and 4th 𝛽-strands, and it was speculated that this hydrophobic surface may facilitate protein interactions (Schubeis et al., 2018). The MSA and the evolutionary conservation predicted by ConSurf indicate that the hydrophobic surface is conserved and extends towards the unstructured C-terminal end to include position 126, 128 and 131 ( Figure 1D ). I126 is found in all 35 sequences investigated here and positions 102 (69% V, 23% I, 9% L), 104 (66% I, 31% V, 3% L), 111 97% L, 3% F), 113 (40% L, 21% V, 9% I), 115 (54% V, 46% I), 128 (94% V, 3% I, 3% L), 131 (77% L, 11% I, 3% V) are occupied by amino acids with hydrophobic side chains ( Figure 1A red :). The conservation of these hydrophobic residues correlates well with the observation that this region is important for Curli assembly. In vivo complementation assays have shown that the C-terminal half of CsgF is needed for Curli assembly and that this region binds the nucleator protein CsgB (Swasthi et al., 2023, Yan et al., 2020, Schubeis et al., 2018). It appears that the 11 residues (between 128-138) at the C-terminal end of CsgF is required for interaction with CsgB and the proper assembly of Curli (Swasthi et al., 2023). D117 is conserved in all 35 sequences of CsgF used in this study however its importance to the structure or function of CsgF is not known.

**Conclusion:** Using a multiple sequence alignment of CsgF sequences from 35 bacterial species, as well as an evolutionary conservation analysis using the ConSurf server, we were able to identify significant amino acid conservation throughout the protein. We were able to correlate several of the conserved positions to published reports describing the regions important for the structure and function of CsgF. However there remains positions of amino acid conservation whose importance is not yet known. A systematic investigation of the conserved positions identified here may help add to our understanding of Curli assembly.
